# Real-world use and survival outcomes of sacituzumab govitecan in metastatic triple-negative breast cancer and hormone receptor-positive/HER2-negative metastatic breast cancer

**DOI:** 10.1038/s41416-026-03346-9

**Published:** 2026-02-05

**Authors:** Aya Elhusseiny Shaaban, Hugo Jourdain, David Desplas, Stéphane Vignot, Mahmoud Zureik, Nadia Haddy

**Affiliations:** 1 EPI-PHARE, French National Agency for Medicine and Health Product Safety (ANSM) and the French National Health Insurance Center (CNAM), Saint-Denis, France; 2 Paris Saclay University, Université Versailles Saint Quentin (UVSQ - EDSP), Orsay, France; 3 UR7509 IRMAIC, université Reims Champagne Ardenne, Reims, France; 4 Département d’oncologie médicale, institut Godinot, Reims, France

**Keywords:** Breast cancer, Risk factors

## Abstract

**Background:**

Sacituzumab govitecan (SG) was granted early access in France as third-line therapy for metastatic triple-negative breast cancer (mTNBC) and hormone receptor-positive/HER2-negative (HR+/HER2–mBC) metastatic breast cancer. This nationwide cohort study assessed its real-world use and survival outcomes.

**Methods:**

Using the French National Health Data System, we included all patients initiating SG between July 1, 2021, and December 31, 2023, with follow-up until June 30, 2024. Patient demographics, comorbidities, and prior treatments were recorded. Overall survival (OS) and time to treatment discontinuation (TTD) were estimated by Kaplan-Meier methods, and multivariable Cox models identified OS prognostic factors.

**Results:**

3653 patients were included: 2527 mTNBC and 1,126 HR+/HER2– mBC, with median ages of 58 and 61.5 years. Median OS was 11.0 months (95%CI: 10.4–11.7) for mTNBC and 11.4 months (95% CI: 10.7–12.4) for HR+/HER2–mBC. One-year survival was 47% and 48% and median TTD of 4.3 and 3.5 months, respectively. Poorer OS was independently associated with inpatient SG initiation and liver/digestive metastases. In mTNBC, additional factors included brain metastases, respiratory disease, tobacco-related hospitalisation, multiple metastatic sites, and prior treatments.

**Conclusion:**

The study highlights SG’s clinical relevance and the challenge of translating trial efficacy into real-world outcomes, reinforcing the need for further investigation of tolerability in broader populations.

## Introduction

Breast cancer is the most commonly diagnosed malignancy worldwide [1], reaching 2.3 million new cases in 2022 [2], and remains the leading cause of cancer-related mortality among women globally, accounting for 670,000 deaths in 2022 [2] and in France, accounting for 12,600 deaths in 2021 [3]. Among its subtypes, triple-negative breast cancer (TNBC), representing approximately 15% of annual breast cancer diagnoses, is one of the most aggressive due to the lack of hormone receptor and HER2 expression, which renders it resistant to targeted therapies to these receptors [4]. Metastatic breast cancer (mBC) remains a major challenge, particularly in the case of mTNBC, where patients generally have a poor prognosis, with a median overall survival (mOS) in metastatic pretreated cases ranging from 11 to 13 months and a five-year OS rate of 11–13% [5–10]. Treatment options for TNBC remain limited, relying primarily on cytotoxic chemotherapy with modest efficacy, particularly in first-line metastatic settings. Although programmed death-ligand 1 (PD-L1)–targeted immunotherapy combined with chemotherapy has shown benefit in 30–40% of PD-L1–positive advanced TNBC cases, mOS remains below one year, highlighting the urgent need for novel, effective therapies [11–13]. In contrast to TNBC, the HR+/HER2− subtype is characterised by the presence of hormone receptors, which allows for targeted therapies such as endocrine treatments and CDK4/6 inhibitors and it is the most frequent breast cancer subtype (70–80%) [14, 15]. While patients with metastatic, pretreated HR + /HER2− breast cancer generally have better five-year survival rates than those with TNBC, reaching approximately 30–34% [16, 17], their mOS remains limited but higher than mTNBC, ranging between 12 and 20 months [18, 19].

Sacituzumab govitecan (SG), a TROP2-targeting antibody-drug conjugate (ADC) commercially known as TRODELVY, has emerged as a promising therapeutic option. ADCs are designed to deliver potent cytotoxic agents directly to cancer cells while minimising systemic toxicity [20–22].

In the Phase I/II IMMU-132 trial [23] and the Phase III ASCENT trial [24–28], SG has demonstrated superior efficacy compared to chemotherapy in patients with mTNBC (median progression-free survival (mPFS) of 5.6 months versus 1.7 months, and mOS of up to 13 months versus 6.7 months). These results are based on the primary analysis, which excluded patients with active cerebral metastases. The observed clinical benefits extended across subgroups, with manageable toxicities such as neutropenia and anemia.

SG has also shown promising efficacy in hormone receptor-positive, HER2-negative (HR+, HER2-) mBC in both Phase I/II Basket [29] and phase III TROPiCS-02 trials [19, 30, 31]. The Basket trial reported an OS of 12 months. The TROPiCS-02 trial further reinforced these findings, demonstrating a mOS of 14.4 months with SG compared to 11.2 months with chemotherapy. In both trials, mPFS was 5.5 months for SG compared to 4.0 months for chemotherapy.

SG received FDA approval in 2020 and EMA/ European Commission approval in November 2021 for mTNBC and in July 2023 for HR+/HER2− mBC. In France, early access was granted in September 2021 for mTNBC, then expanded to HR+/HER2− mBC in February 2023. Clinical trials are essential for drug approval, but real-world data provide complementary insights that may differ from trial outcomes and are important for evaluating long-term outcomes in routine clinical practice. To date, real-world data on mTNBC are limited to small cohorts (from 43 to 409 patients), with median OS ranging from 8.6 to 13.1 months [32–38]. No real-world data are available for the HR+/HER2- population.

The objective of this study is to describe the real-world SG utilisation, patient characteristics, treatment patterns, and survival outcomes in mTNBC and HR+/HER2−mBC treated under the French Early Access Programme using data from the French National Health Data System (SNDS), one of the largest nationwide healthcare administrative databases.

## Methods

This was a descriptive cohort study, with methods and results reported in accordance with the European Society for Medical Oncology’s Guidance for Reporting Oncology Real-World Evidence (ESMO-GROW) [39].

### Data sources

This study was based generally on the French National Health Data System or Système National des Données de Santé (SNDS), which is a comprehensive database encompassing health insurance reimbursement and hospitalization data for over 99% of the French population (approximately 68 million residents). It provides anonymized individual-level data integrating across multiple data sources from which the DCIR (Données de Consommation Inter-Régimes), which includes outpatient reimbursement data, PMSI (Programme de médicalisation des systèmes d’information), which includes the national hospital discharge database containing data on hospital admissions, ICD-10-coded diagnoses, and information on expensive or early-access drugs prescribed during hospital stays. The SNDS has been extensively validated for pharmacoepidemiological research [40–43]. Further details about the SNDS are provided elsewhere [44].

A unique reference tool for early access medications has been developed by our EPI-PHARE team, the Early Access and Expensive Drugs Cohort (AP-MO, *Accès Précoce-Médicaments Onéreux*), which is nested in the SNDS integrating the PMSI data [45]. Expensive and early access medications are identifiable using dosage unit codes *or unité commune de dispensation* (UCD) codes and *liste en sus* (LES, additional funding list) codes. The AP-MO database includes detailed medical indications for each early access treatment delivery.

### Study population

**Fig. 1:**
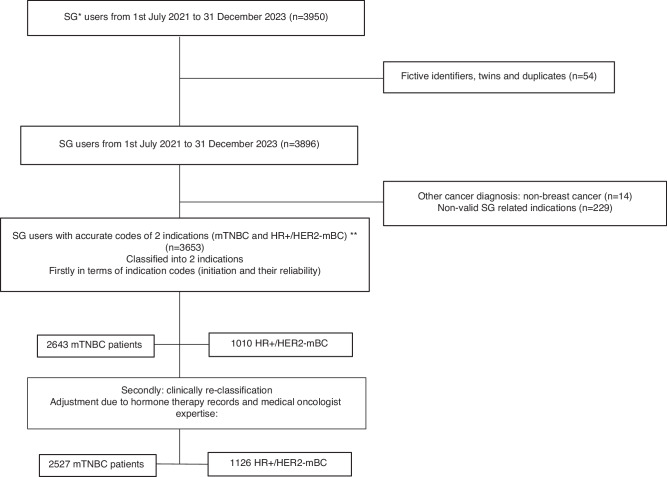
Flowchart of Population Selection and Exclusion Criteria for SG Users in France under the EAPs Framework (July 2021–December 2023). *SG: Sacituzumab Govitecan. **mTNBC: metastatic Triple Negative Breast Cancer. HR+/HER2-mBC: Hormonoreceptor-positive/HER2–negative metastatic Breast Cancer.

Patients were eligible for inclusion if they received at least one delivery of SG (identifying the Anatomical Therapeutic Chemical (ATC) classification system code in conjunction with 2 dosage form codes UCD from the PMSI database) between July 1, 2021 (the date of first authorised SG delivery under early access in France), and December 31, 2023 (last available data at study inclusion time), for codes see Supplementary table 1. Patients were stratified into two distinct cohorts—mTNBC and HR+/HER2− breast cancer—based primarily on medical indication codes (LES codes: *CSACI01* for triple-negative and *CSACI02* for hormone receptor-positive) recorded at the time of SG initiation. A total of 229 patients (6%) were excluded due to inconsistent or incomplete indication coding. LES codes were stable in >95% of cases; in the case of a switch between two indications, the final code is retained in case of a single switch (2%) and the more frequent code is retained in case of multiple switches (1%). Further, 54 duplicates and 14 patients without a recorded breast cancer diagnosis were excluded (See Fig. 1 for flow chart). To enhance classification accuracy and minimise coding-related misclassification, we applied a clinically informed reassignment strategy, similar to a modified per-protocol approach. Patients initially coded as TNBC who received hormone therapy, defined as at least one administration of aromatase inhibitors, tamoxifen, or fulvestrant, within two years after SG initiation (*n* = 116) were reassigned to the HR+/HER2– group. Hormone receptor status cannot newly develop after diagnosis, making TNBC coding incompatible with subsequent hormone therapy. This reassignment was performed in collaboration with an oncology expert (S.V.) and aligned with the SG approval timeline. To assess the robustness of the findings with respect to the study population definition, a sensitivity analysis was performed after excluding these 116 patients.

The final cohort included 3653 patients treated with SG between July 2021 and December 2023. The first SG delivery for each patient was defined as the index date, and follow-up continued until the study cut-off date (June 30, 2024, last day for which data were available at the time of the study) or death, whichever occurred first.

### Patient baseline characteristics

Baseline characteristics of patients included socio-demographic variables such as age (at SG treatment initiation), sex, and French Social Deprivation Index (FDep) (categorised by quintiles). FDep is a marker of socioeconomic status based on the residence area’s median household income, percentage of high-school graduates in the population who are 15 years of age and over, percentage of manual workers in the labour force, and level of unemployment [46]. Comorbid conditions and hospitalisations related to smoking were identified using the “Cartographie des Pathologies et des Dépenses”, a classification tool developed by the French National Health Insurance system (CNAM), which utilises SNDS data through algorithmic disease mapping [47–49]. Additional clinical information was gathered from the previous five years of medical history, including the type of metastasis, number of metastatic sites, prior breast surgeries, and radiotherapy. The treatment history over the preceding year—including hormonal therapy, immunotherapy, and chemotherapy was recorded alongside the initiation of trastuzumab deruxtecan (T-DXd) therapy following SG. The duration of breast cancer was defined as the time from the earliest breast cancer diagnosis code recorded within 10 years before the index date to the index date itself. Duration of metastasis was defined as the time from the earliest metastasis diagnosis code recorded in the 5 years before the index date to the index date. The ICD-10 codes used are listed in the supplementary table 1. Inpatient SG initiation variable together with year of initiation, cancer and metastases duration variables were used as a reliable surrogate or an imperfect proxy for altered performance status, as patients with poorer general condition tend to receive treatment in inpatient clinics [50].

### Statistical analysis

Descriptive statistics were used to summarise patient characteristics by medical indication. Continuous variables were reported as medians with interquartile ranges, while categorical variables were expressed as frequencies and percentages.

#### Outcomes of interest, treatment Exposure and use patterns

The follow-up period was defined as the time from the index date to the date of last SG use prior to death or study end, representing the observation window for survival analyses. Overall survival (OS) was calculated from the index date to death from any cause, with patients censored at the study end if no death was recorded.

Time to Treatment Discontinuation (TTD) and treatment period were both defined as the time from the index date to the earliest occurrence of sacituzumab govitecan (SG) discontinuation, death, or study end. SG discontinuation was defined as the absence of any subsequent delivery of SG within 21 days (reflecting the theoretical dosing interval) plus an additional 60-day washout period, even if SG is resumed afterwards (2% of the total population resumed *n* = 68), unless death occurred earlier. Patients who had not discontinued treatment or died by the study end were censored at that time. Although identically defined, TTD was analysed as a time-to-event outcome, while Treatment Duration served a descriptive purpose.

The number of SG injections was calculated for each patient from the index date to the end of treatment and standardised by individual treatment duration to account for differences in exposure time. Based on the 21-day regimen (two injections per cycle), the number of treatment cycles was estimated accordingly.

#### Survival curves

Kaplan–Meier survival curves with 95% confidence intervals (CIs) were generated to estimate OS and TTD for each medical indication (mTNBC and HR + /HER2− mBC). Prognostic factors associated with mortality were assessed using univariate and multivariate Cox proportional hazards models with 95% confidence intervals for hazard ratios. Stratified analyses were further conducted to assess survival differences across key subgroups: age (≤65 vs. >65 years), number of metastatic sites (<2 vs. ≥2 organs), and presence of brain or liver metastases. No correction for multiple testing was applied, as subgroup analyses were exploratory.

All data extractions from the SNDS were carried out with SAS Enterprise Guide software version 7.15. Survival analyses were performed with R [51] version 3.5.2, using multiple packages, including dplyr [52], ggplot2 [53], and survival [54].

## Results

Between July 2021 and December 2023, 3,653 patients initiated SG as third-line therapy, including 2527 with mTNBC and 1126 with HR+/HER2−BC. (Fig. 1). Among the included patients, 99.7% were women. Detailed demographic and baseline characteristics are summarised in (Table 1).

**Table 1:** Patient characteristics at baseline (SG initiation).

Variable	Overall	mTNBC	HR + /HER2- mBC
N (%)	3653 *n* (%)	2527 *n* (%)	1126 *n* (%)
**Median age in years [IQR]**	59 [50;68]	58 [48;68]	61.5 [53;70]
Age in years			
<50	893 (24.5)	696 (27.5)	197 (17.5)
50–65	1605 (43.9)	1089 (43.1)	516 (45.8)
>65	1155 (31.6)	742 (29.4)	413 (36.7)
**Women**	3642 (99.7)	2520 (99.7)	1122 (99.6)
**Social Deprivation Index (quintile)**			
1st (least disadvantaged)	687 (18.8)	470 (18.6)	217 (19.3)
2^nd^	750 (20.6)	536 (21.2)	214 (19)
3rd	730 (20)	484 (19.2)	246 (21.8)
4th	733 (20)	501 (19.8)	232 (20.6)
5th (most disadvantaged)	644 (17.6)	449 (17.8)	195 (17.3)
Missing	109 (3)	87 (3.4)	22 (2)
**Comorbidity**			
Hypertension	1147 (31.4)	752 (29.8)	395 (35.1)
Hyperlipidemia	456 (12.5)	311 (12.3)	145 (12.9)
Diabetes	376 (10.3)	239 (9.5)	137 (12.2)
Obesity	389 (10.7)	279 (11)	110 (9.8)
Cardiovascular diseases	504 (13.8)	331 (13.1)	173 (15.4)
Respiratory diseases	315 (8.6)	230 (9.1)	85 (7.5)
**Hospitalised for tobacco complications**	391 (10.7)	288 (11.4)	103 (9.1)
**First SG inpatient hospitalization**	289 (7.9)	230 (9.1)	59 (5.2)
**Year of inclusion**			
2021	225 (6.2)	212 (8.4)	13 (1.2)
2022	1280 (35)	1241 (49.1)	39 (3.5)
2023	2148 (58.8)	1074 (42.5)	1074 (95.4)
**Clinical procedures in the past 5 years**			
Radiotherapy	1566 (42.9)	1114 (44.1)	452 (40.1)
Breast surgery	1608 (44)	1398 (55.3)	210 (18.7)
**Previous anticancer treatments in the past year**			
Hormone therapy (AI or AE)^a^	794 (21.7)	321 (12.7)	473 (42.0)
CDK4/6 inhibitors	342 (9.4)	132 (5.2)	210 (18.7)
T-DXd	403 (11)	103 (4.1)	300 (26.6)
Anti PD1/PDL1	440 (12)	411 (16.3)	29 (2.6)
PARP inhibitors	152 (4.2)	133 (5.3)	19 (1.7)
Capecitabine	1280 (35)	929 (36.8)	351 (31.2)
Median number of previous anticancer regimens of these 6 classes [IQR]^**b**^	1 [1;2]	1 [1;2]	1 [1;2]
In categories			
Less than 2^**c**^	2872 (78.6)	2134 (84.4)	738 (65.5)
2 or more	781 (21.4)	393 (15.6)	388 (34.5)
**Median duration of breast cancer (years) [IQR]**	3.4 [1.7;6.3]	2.8 [1.4;5.1]	5.3 [2.9;7.6]
In categories			
<=2	1148 (31.4)	971 (38.4)	177 (15.7)
>2 and <=5	1258 (34.5)	899 (35.6)	359 (31.9)
>5	1247 (34.1)	657 (26)	590 (52.4)
**Metastasis in the past 5 years**			
Median duration of Metastatic disease (years) [IQR]^**b**^	1.4 [0.6;2.8]	1 [0.5;2]	2.5 [1.4;3.9]
In categories			
<1	1129 (30.1)	968 (38.3)	161 (14.3)
1–3	1195 (32.7)	750 (29.7)	445 (39.5)
>3–5	661 (18.1)	258 (10.2)	403 (35.8)
Missing	668 (18.3)	551 (21.8)	117 (10.4)
Sites of metastasis			
Lymph node (not axillary)	984 (26.9)	723 (28.6)	261 (23.2)
Brain	596 (16.3)	449 (17.8)	147 (13.1)
Skin	276 (7.6)	212 (8.4)	64 (5.7)
Bone	1789 (49)	1010 (40)	779 (69.2)
Pulmonary	1206 (33)	861 (34.1)	345 (30.6)
Digestive metastases overall	1329 (36.4)	740 (29.3)	589 (52.3)
o Liver	1200 (32.9)	667 (26.4)	533 (47.3)
o Other digestive	256 (7)	145 (5.7)	111 (9.9)
Other/Not determined	393 (10.8)	258 (10.2)	135 (12)
Median no. of sites involved in metastasis [IQR]^b^	2 [1;3]	2 [1;3]	2 [1;3]
In categories			
Less than 2^**c**^	1680 (46)	1267 (50.1)	413 (36.7)
2 or more	1973 (54)	1260 (49.9)	713 (63.3)

^a^*AI* Aromatase inhibitors or *AE* Antiestrogens.

^b^Patients without documented metastases/prior anticancer regimens were included in the “<2 sites” category.

^**c**^The median number of metastatic sites, duration of metastases and prior anticancer regimens were computed exclusively among patients with at least one documented metastasis/prior treatment.

For mTNBC patients, the median age was 58 years (interquartile range [IQR]: 48–68). Initial SG administration occurred during inpatient hospitalization for 9.1% of patients (*n* = 230). Regarding comorbidities, 13.1% (*n* = 331) had a current or a history of cardiovascular disease, 9.5% (*n* = 239) were diabetic, and 29.8% (*n* = 752) had hypertension. In the five years preceding SG initiation, 44.1% (*n* = 1114) had received radiotherapy, and 55.3% (*n* = 1398) had undergone breast surgery. The median number of metastatic sites was 2 [IQR: 1–3], with brain and liver metastases observed in 17.8% (*n* = 449) and 26.4% (*n* = 667) of cases, respectively. The median duration of breast cancer was 2.8 years [IQR: 1.4–5.1], and of metastases was 1 year [IQR: 0.5–2]. Prior anti-cancer treatments included capecitabine (36.8%, *n* = 929), hormone therapy (12.7%, *n* = 321), CDK4/6 inhibitors (5.2%, *n* = 132), T-DXd (4.1%, *n* = 103), anti-PD1/PDL1 (16.3, *n* = 411), and PARP inhibitors (5.3%, *n* = 133). Most mTNBC patients (84.4%) had received fewer than two prior treatments from these six therapeutic categories in the previous year.

For HR+/HER2−BC patients, the median age was 61.5 years (IQR: 53–70). Initial SG administration occurred during inpatient hospitalization for 5.2% of patients (*n* = 59), 15.4% (*n* = 173) had a current or a history of cardiovascular disease, 12.2% (*n* = 137) were diabetic, and 35.1% (*n* = 395) had hypertension. In the five years preceding SG initiation, 40.1% (*n* = 452) had received radiotherapy and 18.7% (*n* = 210) had undergone breast surgery. The median number of metastatic sites was 2 [IQR: 1–3], with brain metastases observed in 13.1% (*n* = 147), and liver metastases in 47.3% (*n* = 533) of patients. The median duration of breast cancer was 5.3 years [IQR: 2.9–7.6], and the median duration of metastases was 2.5 years [IQR: 1.4–3.9]. Regarding prior anti-cancer treatments within the previous year, 42% (*n* = 473) had received hormone therapy, 31.2% (*n* = 351) had received capecitabine, 18.7% (*n* = 210) had received CDK4/6 inhibitors, 26.6% (*n* = 300) had received T-DXd, 2.6% (*n* = 29) had been treated with anti-PD1/PDL1 inhibitors, and 1.7% (*n* = 19) had received PARP inhibitors. Most HR+/HER2−BC patients (65.5%) had received fewer than two prior treatments from these six therapeutic categories in the previous year.

For mTNBC patients, the median follow-up duration was 9.4 months (IQR 5.2–15.5), and the median treatment duration was 4.3 months (IQR 2.3–7.1). Patients received a median of 5 SG cycles (IQR 2.5–9), i.e., standardised median number of deliveries was 10 (IQR 5–17). Treatment was discontinued in 64.5% (*n* = 1,630), with 2.1% (n = 54) resumed SG use after 81 days. Follow-up characteristics are summarised in (Table 2). For HR+/HER2− mBC patients, the median follow-up duration was 8.4 months (IQR 5.5–11.3), and the median treatment duration was 3.5 months (IQR 2.3–6). Patients received a median of 4 SG cycles (IQR 2.5–7) i.e. standardised median number of deliveries was 8 (IQR 5–14). Treatment was discontinued in 63.3% (*n* = 713), of whom 1.2% (*n* = 14) resumed SG use after more than 81 days.

**Table 2:** Follow up details by groups of indication

Duration in months: Median [IQR]^a^	mTNBC *N*^b^ = 2527	HR + /HER2- mBC *N* = 1126
**Median follow-up time**	9.4 [5.2;15.5]	8.4 [5.5;11.3]
Median follow-up time for those who lived	*n* ^c^= 844 (33.4%) 15 [10.1;22.1]	*n* = 582 (51.7%) 10.1 [8.2;12.8]
Median follow-up time for those who died	*n* = 1683 (66.6%) 6.9 [3.4;11.6]	*n* = 544 (48.3%) 5.3 [2.8;8.4]
**Median treatment time**	4.3 [2.3;7.1]	3.5 [2.3;6]
Median treatment time for who had not discontinued	*n* = 229 (9.1%) 11.7 [8.4;18.5]	*n* = 149 (13.2%) 8.6 [7.1;11]
Median treatment time for who had discontinued	*n* = 2298 (90.9%) 3.8 [2.3;6.4]	*n* = 977 (86.8%) 3.1 [2.3;4.6]
**Use patterns n (%)**		
At least one Switch to T-DXd after SG	395 (15.6)	92 (8.2)
**Median number of SG deliveries/ patient [IQR]**	10 [5;17]	8 [5;14]
In categories *n* (%)		
<=10	1343 (53.1)	724 (64.3)
11–30	960 (38)	373 (33.1)
>30	224 (8.9)	29 (2.6)
**Median number of SG cycles [IQR]**	5 [2.5;9]	4 [2.5;7]

^a^*IQR* Interquartile range.

^b^*N* number total in each indication.

^c^*n* number of patients in each corresponding subgroup.

**Fig. 2:**
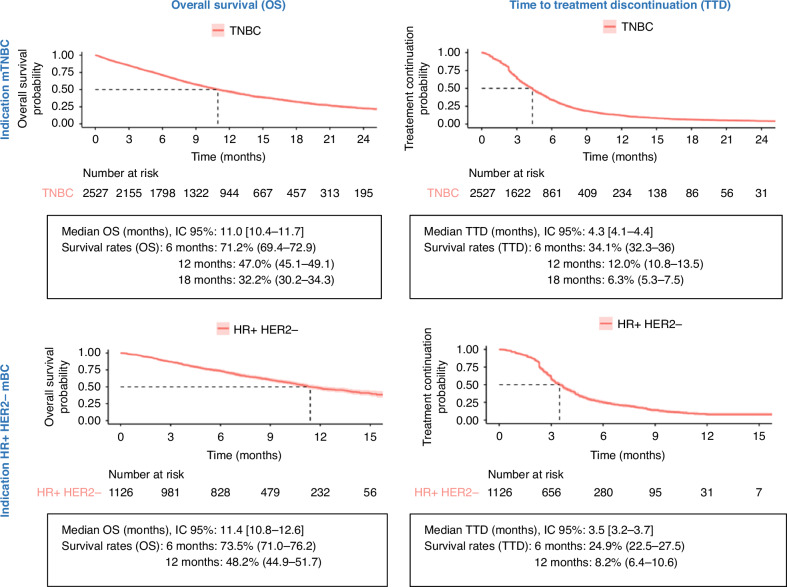
Survival curves for the two indications of SG; mTNBC and HR+/HER2- mBC.

Among mTNBC patients, 66.6% (*n* = 1683) had died by the end of the study. The median OS was 11.0 months (95% CI: 10.4–11.7), with a 1-year survival rate of 46.9% (95% CI: 44.8–48.9). The median TTD was 4.3 months (95% CI: 4.1–4.4), with a 1-year discontinuation rate of 12.0% (95% CI: 10.7–13.4). Among HR+/HER2−BC patients, 48.3% (*n* = 544) had died by the end of the study. The median OS was 11.4 months (95% CI: 10.7–12.6), with a 1-year survival rate of 48.0% [95% CI 44.6–51.4]. The median TTD was 3.5 months [95% CI 3.2–3.7], with a 1-year discontinuation probability of 8.2% [95% CI 6.3–10.5] (Fig. 2).

The sensitivity analysis, performed after excluding the 116 patients reassigned from TNBC to HR + /HER2– showed that OS in the HR+/HER2- cohort changed only slightly from 10.7 (95% CI: 10.0–11.4) to 11.4 (95% CI : 10.7–12.6) months, and TTD remained stable at 3.4 versus 3.5 months (95% CI 3.2–3.7 for both) (Supplementary Table 2).

To further explore the prognostic influence of clinical variables beyond the global survival estimates, stratified Kaplan–Meier was conducted in the TNBC cohort, focusing on the impact of age and metastatic burden on OS and TTD. Older age (>65 years) was associated with longer OS (12.4 vs. 10.5 months). Patients with two or more metastatic sites had worse OS (8.5 vs. 13.8 months) and shorter TTD (3.8 vs. 4.8 months). The presence of brain or liver metastases was linked to a marked reduction in both OS and TTD. Overall, metastatic site involvement—particularly brain and liver—emerged as strong negative prognostic indicators (supplementary fig. 2). Concerning the stratified analysis that was performed in the HR+/HER2– group, patients aged over 65 experienced shorter OS compared to younger counterparts (10.8 vs. 12.2 months), with a non-significant trend toward shorter TTD. A higher number of metastatic sites (≥2) remained a consistent adverse factor, reducing both OS (10.5 vs. 13.8 months) and TTD (3.2 vs. 3.9 months). In contrast to the TNBC group, brain metastases did not impact OS or TTD. Liver metastases, however, remained a strong predictor of poor prognosis, associated with both shorter OS (9.8 vs. 13.6 months) and reduced TTD (3.2 vs. 3.7 months) (Supplementary fig. 3).

Several factors independently associated with worsened survival were identified in multivariable analysis for TNBC group (Table 3): inpatient hospital administration of first dose, HR = 2.20 [95% CI: 1.88–2.57]; respiratory disease, HR = 1.20 [95% CI: 1.01–1.42]; tobacco-related hospitalization, HR = 1.17 [95% CI: 1.01–1.36]; two or more prior treatments across six therapeutic classes, HR = 1.38 [95% CI: 1.21–1.58]; ≥2 metastatic sites, HR = 1.28 [95% CI: 1.09–1.50]; brain metastases, HR = 1.53 [95% CI: 1.35–1.75]; and digestive metastases, including hepatic involvement, HR = 1.48 [95% CI: 1.31–1.67].

**Table 3:** Univariate and adjusted Overall survival Hazard ratios for mTNBC group.

Variable	mTNBC	Alive/censured	Died	Univariate	Adjusted
*N* (%)	2527 (69.2)	844 (33.4)	1683 (66.6)	HR [95% CI]	HR [95% CI]
**Age (years)**					
<50	696 (27.5)	196 (23.2)	500 (29.7)	1	1
50–65	1089 (43.1)	371 (44)	718 (42.7)	0.81 [0.72–0.91]	0.84 [0.74–0.94]
>65	742 (29.4)	277 (32.8)	465 (27.6)	0.77 [0.68–0.87]	0.9 [0.78–1.03]
**Social Deprivation Index**^**a**^					
Less disadvantaged (1st, 2nd)	1006 (39.8)	347 (41.1)	659 (39.2)	1	1
More disadvantaged (3rd, 4th, 5th)	1434 (56.8)	467 (55.3)	967 (57.5)	1.02 [0.92–1.13]	0.99 [0.9–1.09]
**Year of inclusion**					
2021	212 (8.4)	24 (2.8)	188 (11.2)	1.27 [1.08–1.51]	1.35 [1.14–1.61]
2022	1241 (49.1)	291 (34.5)	950 (56.4)	1 [0.9–1.12]	1.08 [0.96–1.2]
2023	1074 (42.5)	529 (62.7)	545 (32.4)	1	
**First SG in inpatient hospitalization**	230 (9.1)	27 (3.2)	203 (12.1)	2.98 [2.57–3.45]	2.18 [1.86–2.56]
**Comorbidity in the past 5 years**					
Hypertension	752 (29.8)	255 (30.2)	497 (29.5)	1 [0.9–1.12]	1.04 [0.92–1.17]
Hyperlipidemia	311 (12.3)	112 (13.3)	199 (11.8)	0.94 [0.81–1.09]	1.03 [0.88–1.22]
Diabetes	239 (9.5)	83 (9.8)	156 (9.3)	0.96 [0.82–1.13]	1 [0.83-1.2]
Obesity	279 (11)	88 (10.4)	191 (11.3)	1.04 [0.9–1.21]	0.95 [0.82–1.12]
CVD	331 (13.1)	108 (12.8)	223 (13.3)	1.09 [0.95–1.26]	1.02 [0.88–1.19]
Respiratory diseases	230 (9.1)	65 (7.7)	165 (9.8)	1.28 [1.09–1.5]	1.2 [1.02–1.42]
**Hospitalised for tobacco complications**	288 (11.4)	79 (9.4)	209 (12.4)	1.24 [1.08–1.44]	1.19 [1.02–1.38]
**Nb of previous treatments of 6 classes one year before date index**					
Less than 2^**b**^	2134 (84.4)	726 (86)	1408 (83.7)	1	1
2 or more	393 (15.6)	118 (14)	275 (16.3)	1.38 [1.21–1.57]	1.35 [1.18–1.55]
**Metastases in 5 past years**					
**Duration of Metastases (years)**^**b**^					
<1	968 (38.3)	264 (31.3)	704 (41.8)	1	1
1-3	750 (29.7)	266 (31.5)	484 (28.8)	0.77 [0.68–0.86]	0.76 [0.67–0.85]
>3-5	258 (10.2)	97 (11.5)	161 (9.6)	0.73 [0.61–0.86]	0.72 [0.61–0.86]
**Number of sites involved in metastases**					
Less than 2^**b**^	1267 (50.1)	524 (62.1)	743 (44.1)	1	1
2 or more	1260 (49.9)	320 (37.9)	940 (55.9)	1.71 [1.55–1.88]	1.28 [1.09–1.51]
**Sites of metastasis**					
Ganglionic (not axillary)	723 (28.6)	226 (26.8)	497 (29.5)	1.08 [0.97–1.2]	0.91 [0.81–1.02]
Brain	449 (17.8)	85 (10.1)	364 (21.6)	1.82 [1.62–2.05]	1.52 [1.34–1.74]
Skin	212 (8.4)	61 (7.2)	151 (9)	1.09 [0.92–1.29]	1.01 [0.85–1.21]
Bone	1010 (40)	298 (35.3)	712 (42.3)	1.37 [1.24–1.51]	1.13 [1–1.28]
Pulmonary	861 (34.1)	219 (25.9)	642 (38.1)	1.39 [1.26–1.53]	1.1 [0.98–1.24]
Liver	667 (26.4)	147 (17.4)	520 (30.9)	1.77 [1.6–1.96]	1.46 [1.3–1.65]
Other digestive	145 (5.7)	27 (3.2)	118 (7)	2.05 [1.7–2.47]	1.5 [1.23–1.83]
Other/NP	258 (10.2)	69 (8.2)	189 (11.2)	1.29 [1.11–1.5]	1.01 [0.86–1.2]

^a^Patients without documented metastases/prior anticancer regimens were included in the “<2 sites” category.

^b^Missing-data categories of Variables, such as the duration of metastasis (18%) and the social deprivation index (3%) were retained in the cox models to maintain statistical power but were not shown in this table.

For the HR+/HER2−BC group (Table 4), factors independently associated with worsened survival were identified in multivariable analysis: age >65 years compared to <50 years, HR = 1.25 [95% CI: 1.04–1.50]; inpatient administration of first dose, HR = 3.00 [95% CI: 2.12–4.18]; and digestive metastases, including hepatic involvement, HR = 1.51 [95% CI: 1.22–1.87]. No significant associations were observed for the number of metastatic sites, brain metastases, or metastatic disease duration. Skin metastases demonstrated a modest but statistically significant association with reduced OS (HR = 1.43 [95% CI: 1.02–2.00]).

**Table 4:** Univariate and adjusted Overall survival Hazard Ratios for HR + /HER2- mBC group.

Variable	HR + /HER2- mBC	Alive /censured	Died	Univariate	Adjusted
*N* (%)	1126 (30.8)	582 (51.7)	544 (48.3)	HR [95% CI]	HR [95% CI]
**Age (years)**					
<50	197 (17.5)	109 (18.7)	88 (16.2)	1	1
50 - 65	516 (45.8)	276 (47.4)	240 (44.1)	1.01 [0.79–1.29]	1.07 [0.83–1.37]
>65	413 (36.7)	197 (33.8)	216 (39.7)	1.21 [0.94–1.55]	1.31 [1–1.71]
**Social Deprivation Index**^**a**^					
Less disadvantaged (1st, 2nd)	431 (38.3)	212 (36.4)	219 (40.3)	1	1
More disadvantaged (3rd, 4th, 5th)	673 (59.7)	355 (61)	318 (58.5)	0.92 [0.78–1.09]	0.85 [0.71–1.01]
**First SG in inpatient hospitalization**	59 (5.2)	16 (2.7)	43 (7.9)	3.03 [2.22–4.15]	2.98 [2.12–4.18]
**Comorbidity in the past 5 years**					
Hypertension	395 (35.1)	214 (36.8)	181 (33.3)	0.94 [0.78–1.12]	0.84 [0.68–1.05]
Hyperlipidemia	145 (12.9)	81 (13.9)	64 (11.8)	0.91 [0.7–1.18]	0.96 [0.72–1.28]
Diabetes	137 (12.2)	78 (13.4)	59 (10.8)	0.92 [0.7–1.2]	0.91 [0.68–1.22]
Obesity	110 (9.8)	61 (10.5)	49 (9)	0.92 [0.68–1.23]	0.85 [0.63–1.16]
CVD	173 (15.4)	81 (13.9)	92 (16.9)	1.23 [0.98–1.54]	1.14 [0.89–1.45]
Respiratory diseases	85 (7.5)	36 (6.2)	49 (9)	1.28 [0.95–1.71]	1.26 [0.93–1.71]
**Hospitalised for tobacco complications**	103 (9.1)	51 (8.8)	52 (9.6)	1.09 [0.82–1.46]	1.14 [0.85–1.53]
**Nb of previous treatments of 5 categories**					
Less than 2^**b**^	738 (65.5)	389 (66.8)	349 (64.2)	1	1
2 or more	388 (34.5)	193 (33.2)	195 (35.8)	1.07 [0.9–1.27]	0.97 [0.81–1.16]
**Metastases in 5 past years**					
**Duration of Metastases (years)**^**a**^					
<1	161 (14.3)	78 (13.4)	83 (15.3)	1	1
1–3	445 (39.5)	221 (38)	224 (41.2)	0.91 [0.71–1.17]	0.86 [0.66–1.12]
>3–5	403 (35.8)	216 (37.1)	187 (34.4)	0.86 [0.66–1.11]	0.81 [0.61–1.07]
**Number of sites involved in metastases**					
Less than 2^**b**^	413 (36.7)	242 (41.6)	171 (31.4)	1	1
2 or more	713 (63.3)	340 (58.4)	373 (68.6)	1.48 [1.23–1.78]	1.22 [0.91–1.65]
**Sites of metastasis**					
Ganglionic (not axillary)	261 (23.2)	133 (22.9)	128 (23.5)	0.99 [0.82–1.21]	0.84 [0.68–1.05]
Brain	147 (13.1)	68 (11.7)	79 (14.5)	1.25 [0.98–1.58]	1.11 [0.86–1.42]
Skin	64 (5.7)	24 (4.1)	40 (7.4)	1.5 [1.09–2.08]	1.43 [1.02–1.99]
Bone	779 (69.2)	391 (67.2)	388 (71.3)	1.23 [1.02–1.48]	1.18 [0.91–1.52]
Pulmonary	345 (30.6)	169 (29)	176 (32.4)	1.16 [0.97–1.39]	1.03 [0.83–1.26]
Liver	533 (47.3)	239 (41.1)	294 (54)	1.5 [1.27–1.78]	1.51 [1.22–1.87]
Other digestive	111 (9.9)	48 (8.2)	63 (11.6)	1.46 [1.12–1.89]	1.4 [1.06–1.86]
Other/NP	135 (12)	64 (11)	71 (13.1)	1.14 [0.89–1.46]	1.02 [0.79–1.33]

^a^Patients without documented metastases/prior anticancer regimens were included in the “<2 sites” category.

^**b**^Missing-data categories of Variables, such as the duration of metastasis (18%) and the social deprivation index (3%) were retained in the Cox models to maintain statistical power but were not shown in this table.

## Discussion

This nationwide study represents the largest real-world analysis to date evaluating SG utilisation in both mTNBC and HR+/HER2− mBC. Median OS was 11.0 months for mTNBC and 11.4 months for HR+/HER2- mBC, with 1-year survival rates of 47.0% and 48.0%, respectively. Median TTDs were 4.3 and 3.5 months, respectively. Regarding the reassignment procedure, the robustness of the OS and TTD estimates in the sensitivity analysis confirmed that correcting this coding-based misclassification did not affect outcome patterns and that the integrity of the findings was maintained.

Compared with pivotal clinical trials, survival outcomes in our cohort were slightly lower. In mTNBC, the Phase II IMMU-132-01 trial (*n* = 108) reported a median OS of 13.0 months [23], and in the Phase III ASCENT (n = 235), the median OS was 12.1 months with the SG arm [24]. Six-and twelve-month survival rates in the ASCENT trial were 78.5% and 51.3% compared to 71.2% and 46.0% in the mTNBC group. However, clinical trials differ from real-world practice, notably due to the underrepresentation of elderly patients and those with significant comorbidities. In the Phase II and ASCENT trials, patients were generally younger, with reported median ages of 54 and 55 years, respectively, and individuals with active brain metastases were excluded from the primary analysis. In contrast, in our mTNBC cohort, the median age was 58 years, and 17.8% of patients had brain metastases.

In HR+/HER2− mBC, survival outcomes in our cohort were also slightly lower than those observed in the Basket Phase I/II study (*n* = 54, mOS=12 months) [29] and the TROPiCS‑02 Phase III trial (*n* = 268), where SG improved median OS to 14.4 months [19]. Patients in the Phase II and TROPiCS‑02 trials were generally younger, with median ages of 54 and 56 years, respectively, compared to 61.5 years in our cohort. Moreover, 12.8% of our patients had brain metastases, whereas only those with previously treated and stable brain metastases were eligible in TROPiCS‑02.

In our study, older patients show better overall survival in mTNBC (50-56 years vs. <50 years, HR 95% CI: 0.84 [0.74–0.94]), contrary to the poorer outcomes seen with increasing age in hormone HR+/HER2- mBC cases (>65 years vs. <50 years, HR 95% CI: 0.84 [0.74–0.94]). Previous literature shows no consistent conclusion regarding the prognostic role of age in TNBC. Some studies report higher mortality in very young women in both luminal subtypes and TNBC, whereas others describe poorer outcomes among the very elderly [55, 56]. Overall, results remain heterogeneous and depend in part on biological subtypes, including genetic mutations [7, 57]. In addition, real-world treatment patterns may also contribute to the paradox we observed. Younger women may initiate SG earlier because of more rapid disease progression [58]. Accordingly, our finding is best interpreted as an observed association underlying biological heterogeneity and clinical treatment factors rather than evidence of a protective age in TNBC.

Previous real-world studies evaluating SG have been limited by small sample sizes (from 43 to 409 patients) and have focused almost exclusively on the mTNBC [32–38]. Despite variations in patient characteristics—including metastatic burden, performance status, and prior treatment exposure—SG has consistently demonstrated clinical benefit across cohorts from the UK, France, the U.S., Italy, and Germany. Median OS ranged from 8.6 to 13.1 months, with more favourable outcomes observed in patients receiving SG earlier in the treatment sequence, those with better performance status, and those without brain or visceral metastases. The UK cohort (*n* = 126) reported a median OS of 8.7 months, likely influenced by a poorer baseline performance status, with brain metastases present in 18% of patients [37]. Similarly, the French study (*n* = 99) observed the lowest OS (8.6 months), attributed to a high metastatic burden (66% with ≥3 metastatic sites) and a short follow-up duration [32]. Two U.S.-based cohorts (*n* = 230 and 115) reported OS of 10.0 and 9.6 months, with heterogeneous profiles in terms of metastatic sites and prior therapy [33, 38]. A third and larger U.S. study (*n* = 409) observed a median OS of 11.3 months [35], closely aligning with the outcomes in our cohort, particularly with respect to follow-up time and SG exposure. In contrast, Italian (*n* = 57) [34] and German (*n* = 43) [36] cohorts reported higher OS estimates (12.4 and 13.1 months, respectively), possibly due to more frequent exposure to surgery and radiotherapy. Notably, across all studies, liver and brain metastases consistently emerged as adverse prognostic indicators.

### Metastatic disease characteristics

In the ASCENT trial, a secondary subgroup analysis including the 32 patients with brain metastases (total n = 267) resulted in a slight decrease in mOS to 11.8 months (12.1 months in the primary analysis excluding those with stable brain metastases) and mPFS to 4.8 months (vs. 5.6 months in the primary analysis) [26]. In our study, the survival difference was more remarkable due to statistical power, where brain metastases were significantly associated with shorter OS (HR: 1.52; 95% CI: 1.34–1.74) and shorter TTD (HR: 1.30; 95% CI: 1.17–1.45). In contrast, a real-world U.S. study (*n* = 115) [33] found no significant difference in OS between patients with (21.7%) and without brain metastases in patients taking SG. This is likely due to the small sample size, resulting in low statistical power.

In the Phase 3 ASCENT trial [26], the median time from the diagnosis of metastatic disease to initiation of SG was 15.8 months, with a longer duration of 22.5 months for HR+ or HER2+ breast cancer (representing 30% of the cohort). These findings are consistent with our real-world results, where the median duration of metastatic disease before SG initiation was 16.8 months overall with 12 months in the TNBC group and 30 months in the HR+/HER2− group.

### Subgroup analyses

In the ASCENT trial, patients with an initial TNBC diagnosis had a slightly lower median OS compared to those initially classified as HR+ and/or HER2+ (12.1 vs. 12.4 months in the SG arm) [24]. A similar trend was observed in our study, with median OS of 11.0 months in the TNBC group and 11.4 months in the HR + /HER2− group.

Liver metastases were associated with poorer survival in both studies. ASCENT reported a median OS of 9.4 months in patients with liver involvement versus 14.5 months without. Our results showed a consistent negative impact of liver (and digestive) metastases on overall survival.

In the TROPiCS-02 trial, a higher number of prior treatments was linked to worse OS in HR+/HER2− mBC. In contrast, this factor was not significant in our HR+ population.

Additionally, in the TNBC cohort, smoking-related hospitalisations and respiratory comorbidities were both independently associated with poorer survival. These findings support prior evidence that smoking is an adverse prognostic factor in cancer patients [59].

The operational definition of TTD used in our study (21 days corresponding to the theoretical SG dosing interval plus a 60-day washout period) was chosen to avoid misclassifying temporary delays as treatment discontinuation. Similar discontinuation-defined gaps (60–120 days) have been used in real-world studies relying on claims data to measure persistence with targeted immunotherapies and antibody–drug conjugates [60–62]. A 60-day grace window provides a reasonable balance between sensitivity and specificity, allowing for delays related to toxicity, intervening events, or scheduling issues while still capturing true discontinuation events. Nonetheless, TTD remains sensitive to the operational definition applied, and this approach may slightly under- or overestimate true discontinuation in individual cases. This is inherent to claims-based analyses and should be considered when comparing TTD with clinical trial endpoints such as PFS.

The main strength of our study is that it constitutes the largest real-world cohort to date (*n* = 3653) evaluating SG, made possible by the exhaustive coverage of the French SNDS database. This allowed for a comprehensive assessment of hospitalisations and access to OS and TTD data. Additionally, the augmented Early Access and Expensive Drugs Cohort (APMO), which includes documented treatment indications, enabled analysis aligned with randomised clinical trial criteria. However, several limitations must be acknowledged. Determining treatment indications can be challenging, as our algorithm relied on manually entered data, potentially subject to misclassification. We mitigated this risk through the use of dominant coding patterns and reclassification validated regarding patient characteristics and expert review. Moreover, the conducted subgroup analyses were exploratory and performed without correction for multiple testing; therefore, these results should be interpreted with caution. Furthermore, the SNDS database contains reimbursed healthcare data at the national level but does not provide clinical details such as radiological progression, objective response, or the clinical rationale for treatment discontinuation. Although subsequent treatments and some toxicity-related events (hospitalisations or specific supportive medications) can be identified, these events cannot reliably indicate whether discontinuation was due to progression, toxicity, patient recovery, or physician/patient decision. Clinical PFS and ORR cannot be estimated in our database. However, the combined analysis of OS and TTD provides meaningful insight into real-world treatment outcomes. Similarly, the ECOG performance status score was not directly available in the dataset; however, it was approximated using the setting of first treatment administration, with inpatient initiation serving as a proxy for poorer general condition, as patients with lower functional status are more likely to require hospitalization at treatment onset.

## Conclusions

This is the largest study to date providing insights into the effectiveness of SG. Median OS was longer than that reported in smaller real-world studies, yet remained shorter than in clinical trials, likely reflecting broader patient heterogeneity and differences in selection and treatment context. Further analyses focusing on treatment tolerability are warranted to support the optimisation of SG use in clinical practice.

## Supplementary information

Supplementary materials

ESMO GROW reporting guidelines checklist and score

## Data Availability

In accordance with data protection legislation and the French regulation, the authors are not allowed to release or make public the data from the SNDS. However, any person or structure, public or private, for-profit or nonprofit, is able to access SNDS data in order to carry out a study, research, or an evaluation in the public interest, upon authorisation from the CNIL, via the French Health Data Hub (https://www.health-data-hub.fr/).

## References

[1] Sung H, Ferlay J, Siegel RL, Laversanne M, Soerjomataram I, Jemal A, et al. Global Cancer Statistics 2020: GLOBOCAN Estimates of Incidence and Mortality Worldwide for 36 Cancers in 185 Countries. CA: A Cancer J Clin. 2021;71:209–49. 10.3322/caac.21660 33538338

[2] WHO. Breast cancer. https://www.who.int/news-room/fact-sheets/detail/breast-cancer (accessed 15 June 2025).

[3] https://www.e-cancer.fr/Expertises-et-publications/Catalogue-des-publications/Panorama-des-cancers-en-France-edition-2024 Panorama des cancers en France - édition 2024 - Ref: PANOKFR2024(accessed 28 Nov 2024).

[4] Triple-negative Breast Cancer | Details, Diagnosis, and Signs. https://www.cancer.org/cancer/types/breast-cancer/about/types-of-breast-cancer/triple-negative.html (accessed 21 May 2025).

[5] Cancer Statistics. SEER. https://seer.cancer.gov/statistics/index.html (accessed 21 May 2025).

[6] den Brok WD, Speers CH, Gondara L, Baxter E, Tyldesley SK, Lohrisch CA. Survival with metastatic breast cancer based on initial presentation, de novo versus relapsed. Breast Cancer Res Treat. 2017;161:549–56. 28000014 10.1007/s10549-016-4080-9

[7] Zagami P, Carey LA. Triple negative breast cancer: Pitfalls and progress. npj Breast Cancer. 2022;8:95. 35987766 10.1038/s41523-022-00468-0 PMC9392735

[8] Skinner KE, Haiderali A, Huang M, Schwartzberg LS. Real-world effectiveness outcomes in patients diagnosed with metastatic triple-negative breast cancer. Future Oncol. 2021;17:931–41. 33207944 10.2217/fon-2020-1021

[9] O'Reilly D, Sendi MA, Kelly CM. Overview of recent advances in metastatic triple negative breast cancer. World J Clin Oncol. 2021;12:164–82. 33767972 10.5306/wjco.v12.i3.164 PMC7968109

[10] Kesireddy M, Elsayed L, Shostrom VK, Agarwal P, Asif S, Yellala A, et al. Overall Survival and Prognostic Factors in Metastatic Triple-Negative Breast Cancer: A National Cancer Database Analysis. Cancers. 2024;16:1791. 38791870 10.3390/cancers16101791 PMC11120599

[11] Li Y, Zhang H, Merkher Y, Chen L, Liu N, Leonov S, et al. Recent advances in therapeutic strategies for triple-negative breast cancer. J Hematol Oncol **15**, 1212022). 10.1186/s13045-022-01341-0 PMC9422136 36038913

[12] Schmid P, Rugo HS, Adams S, Schneeweiss A, Barrios CH, Iwata H, et al. Atezolizumab plus nab-paclitaxel as first-line treatment for unresectable, locally advanced or metastatic triple-negative breast cancer (IMpassion130): updated efficacy results from a randomised, double-blind, placebo-controlled, phase 3 trial. Lancet Oncol. 2020;21:44–59. 31786121 10.1016/S1470-2045(19)30689-8

[13] Cortes J, Cescon DW, Rugo HS, Nowecki Z, Im S-A, Yusof MM, et al. Pembrolizumab plus chemotherapy versus placebo plus chemotherapy for previously untreated locally recurrent inoperable or metastatic triple-negative breast cancer (KEYNOTE-355): a randomised, placebo-controlled, double-blind, phase 3 clinical trial. Lancet. 2020;396:1817–28. 33278935 10.1016/S0140-6736(20)32531-9

[14] Understanding how Estrogen Receptor Positive (ER+) breast cancers evade the immune system - National Breast Cancer Foundation (NBCF) | Donate Online. https://nbcf.org.au/project/understanding-how-estrogen-receptor-positive-er-breast-cancers-evade-the-immune-system/#:~:text=Estrogen%20Receptor%20Positive%20(ER%2B)%20breast%20cancer%20is%20the%20most,of%20all%20breast%20cancer%20cases.https://kusuri.ansm-intra.fr/prodIntra/ (accessed 3 Jun2025).

[15] Statut des récepteurs hormonaux du cancer du sein. https://www-breastcancer-org.translate.goog/pathology-report/hormone-receptor-status?_x_tr_sl=en&_x_tr_tl=fr&_x_tr_hl=fr&_x_tr_pto=rq&_x_tr_hist=true (accessed 6 June 2025).

[16] Cardoso F, Paluch-Shimon S, Senkus E, Curigliano G, Aapro MS, André F, et al. 5th ESO-ESMO international consensus guidelines for advanced breast cancer (ABC 5). Ann Oncol. 2020;31:1623–49. 32979513 10.1016/j.annonc.2020.09.010 PMC7510449

[17] Surveillance, Epidemiology, and End Results Program. SEER. https://seer.cancer.gov/index.html (accessed 21 May 2025).

[18] Verma S, Miles D, Gianni L, Krop IE, Welslau M, Baselga J, et al. Trastuzumab Emtansine for HER2-Positive Advanced Breast Cancer. N Engl J Med. 2012;367:1783–91. 23020162 10.1056/NEJMoa1209124 PMC5125250

[19] Rugo HS, Bardia A, Marmé F, Cortés J, Schmid P, Loirat D, et al. Overall survival with sacituzumab govitecan in hormone receptor-positive and human epidermal growth factor receptor 2-negative metastatic breast cancer (TROPiCS-02): a randomised, open-label, multicentre, phase 3 trial. Lancet. 2023;402:1423–33. 37633306 10.1016/S0140-6736(23)01245-X

[20] Spring LM, Nakajima E, Hutchinson J, Viscosi E, Blouin G, Weekes C, et al. Sacituzumab govitecan for metastatic triple-negative breast cancer: clinical overview and management of potential toxicities. Oncologist. 2021;26:827–34. 34176192 10.1002/onco.13878 PMC8488774

[21] Moy B, Rumble RB, Come SE, Davidson NE, Di Leo A, Gralow JR, et al. Chemotherapy and Targeted Therapy for Patients With Human Epidermal Growth Factor Receptor 2–Negative Metastatic Breast Cancer That is Either Endocrine-Pretreated or Hormone Receptor–Negative: ASCO Guideline Update. JCO. 2021;39:3938–58. 10.1200/JCO.21.01374 34324366

[22] Goldenberg DM, Stein R, Sharkey RM. The emergence of trophoblast cell-surface antigen 2 (TROP-2) as a novel cancer target. Oncotarget. 2018;9:28989–29006. 29989029 10.18632/oncotarget.25615 PMC6034748

[23] Bardia A, Mayer IA, Vahdat LT, Tolaney SM, Isakoff SJ, Diamond JR, et al. Sacituzumab Govitecan-hziy in Refractory Metastatic Triple-Negative Breast Cancer. N Engl J Med. 2019;380:741–51. 30786188 10.1056/NEJMoa1814213

[24] O’Shaughnessy J, Brufsky A, Rugo HS, Tolaney SM, Punie K, Sardesai S, et al. Analysis of patients without and with an initial triple-negative breast cancer diagnosis in the phase 3 randomized ASCENT study of sacituzumab govitecan in metastatic triple-negative breast cancer. Breast Cancer Res Treat. 2022;195:127–39. 35545724 10.1007/s10549-022-06602-7 PMC9374646

[25] Carey LA, Loirat D, Punie K, Bardia A, Diéras V, Dalenc F, et al. Sacituzumab govitecan as second-line treatment for metastatic triple-negative breast cancer—phase 3 ASCENT study subanalysis. npj Breast Cancer. 2022;8:72. 35680967 10.1038/s41523-022-00439-5 PMC9184615

[26] Hurvitz SA, Bardia A, Punie K, Kalinsky K, Carey LA, Rugo HS, et al. Subgroup analyses from the phase 3 ASCENT study of sacituzumab govitecan in metastatic triple-negative breast cancer. npj Breast Cancer. 2024;10:33. 38664404 10.1038/s41523-024-00635-5 PMC11045722

[27] Bardia A, Hurvitz SA, Tolaney SM, Loirat D, Punie K, Oliveira M, et al. Sacituzumab Govitecan in Metastatic Triple-Negative Breast Cancer. N Engl J Med. 2021;384:1529–41. 33882206 10.1056/NEJMoa2028485

[28] Bardia A, Rugo HS, Tolaney SM, Loirat D, Punie K, Oliveira M, et al. Final Results From the Randomized Phase III ASCENT Clinical Trial in Metastatic Triple-Negative Breast Cancer and Association of Outcomes by Human Epidermal Growth Factor Receptor 2 and Trophoblast Cell Surface Antigen 2 Expression. JCO. 2024;42:1738–44. 10.1200/JCO.23.01409 PMC11107894 38422473

[29] Kalinsky K, Diamond JR, Vahdat LT, Tolaney SM, Juric D, O'Shaughnessy J, et al. Sacituzumab govitecan in previously treated hormone receptor-positive/HER2-negative metastatic breast cancer: final results from a phase I/II, single-arm, basket trial. Ann Oncol. 2020;31:1709–18. 32946924 10.1016/j.annonc.2020.09.004

[30] Rugo HS, Bardia A, Marmé F, Cortes J, Schmid P, Loirat D, et al. Sacituzumab Govitecan in Hormone Receptor–Positive/Human Epidermal Growth Factor Receptor 2–Negative Metastatic Breast Cancer. JCO. 2022;40:3365–76. 10.1200/JCO.22.01002 36027558

[31] Rugo HS, Bardia A, Tolaney SM, Arteaga C, Cortes J, Sohn J, et al. TROPiCS-02: A Phase III Study Investigating Sacituzumab Govitecan in the Treatment of HR+/HER2- Metastatic Breast Cancer. Future Oncol. 2020;16:705–15. 32223649 10.2217/fon-2020-0163

[32] De Moura A, Loirat D, Vaillant S, Korbi S, Kiavue N, Bello Roufai D, et al. Sacituzumab govitecan in metastatic triple-negative breast cancer patients treated at Institut Curie Hospitals: efficacy, safety, and impact of brain metastases. Breast Cancer. 2024;31:572–80. 38600429 10.1007/s12282-024-01565-7 PMC11194191

[33] Alaklabi S, Roy AM, Zagami P, Chakraborty A, Held N, Elijah J, et al. Real-world clinical outcomes with sacituzumab govitecan in metastatic triple-negative breast cancer. JCO Oncol Pr. 2025;21:620–28. 10.1200/OP.24.00242 PMC12165508 39353151

[34] Caputo R, Buono G, Piezzo M, Martinelli C, Cianniello D, Rizzo A, et al. Sacituzumab Govitecan for the treatment of advanced triple negative breast cancer patients: a multi-center real-world analysis. Front Oncol **14** (2024) 10.3389/fonc.2024.1362641. 10.3389/fonc.2024.1362641 PMC11002149 38595817

[35] Gorantla V, Choski R, Kudrik F, et al. Real-world sacituzumab govitecan treatment patterns and outcomes in second-line or later metastatic triple negative breast cancer: leveraging electronic health records and manual curation of a US database.Presented at: 42nd Annual Miami Breast Cancer Conference. March 6-9, 2025; Miami, Florida. Poster 31. U.S. (2025) https://www.targetedonc.com/view/sacituzumab-govitecan-shows-consistent-efficacy-in-real-world-mtnbc (accessed 2 May 2025).

[36] Reinisch M, Bruzas S, Spoenlein J, Shenoy S, Traut A, Harrach H, et al. Safety and effectiveness of sacituzumab govitecan in patients with metastatic triple-negative breast cancer in real-world settings: first observations from an interdisciplinary breast cancer centre in Germany. Ther Adv Med Oncol. 2023;15:175883592312004542023. 10.1177/17588359231200454 PMC10542232 37789989

[37] Hanna D, Merrick S, Ghose A, Devlin MJ, Yang DD, Phillips E, et al. Real world study of sacituzumab govitecan in metastatic triple-negative breast cancer in the United Kingdom. Br J Cancer. 2024;130:1916–20. 38658782 10.1038/s41416-024-02685-9 PMC11183215

[38] Kalinsky K, Spring L, Yam C, Bhave MA, Ntalla I, Lai C, et al. Real-world use patterns, effectiveness, and tolerability of sacituzumab govitecan for second-line and later-line treatment of metastatic triple-negative breast cancer in the United States. Breast Cancer Res Treat. 2024;208:203–14. 38904892 10.1007/s10549-024-07412-9 PMC11452463

[39] Castelo-Branco L, Pellat A, Martins-Branco D, Valachis A, Derksen JWG, Suijkerbuijk KPM, et al. ESMO Guidance for Reporting Oncology Real-World Evidence (GROW). ESMO Real World Data and Digit Oncol **1** (1000032023). 10.1016/j.esmorw.2023.10.001 PMC12836650 41647770

[40] Jourdain H, Meglio AD, Mansouri I, Desplas D, Zureik M, Haddy N. Use and outcomes of trastuzumab deruxtecan in HER2-positive and HER2-low metastatic breast cancer in a real-world setting: a nationwide cohort study. ESMO Open **9** (2024) 10.1016/j.esmoop.2024.104083. 10.1016/j.esmoop.2024.104083 PMC11697054 39662227

[41] Weill A, Nguyen P, Labidi M, Cadier B, Passeri T, Duranteau L, et al. Use of high dose cyproterone acetate and risk of intracranial meningioma in women: cohort study. BMJ (**372**, n37). 10.1136/bmj.n37 33536184

[42] Botton J, Jabagi MJ, Bertrand M, Baricault B, Drouin J, Le Vu S, et al. Risk for Myocardial Infarction, Stroke, and Pulmonary Embolism Following COVID-19 Vaccines in Adults Younger Than 75 Years in France. Ann Intern Med. 2022;175:1250–57. 35994748 10.7326/M22-0988 PMC9425709

[43] Jourdain H, Albin N, Monard A, Desplas D, Zureik M, Haddy N Trastuzumab Deruxtecan in Human Epidermal Growth Factor Receptor 2-Positive Metastatic Gastric Cancer in a Real-World Setting: A Nationwide Cohort Study. Clin Transl Gastroenterol (10.14309/ctg.00000000000007732022). 10.14309/ctg.0000000000000773 PMC11671096 39373321

[44] Tuppin P, Rudant J, Constantinou P, Gastaldi-Ménager C, Rachas A, de Roquefeuil L, et al. Value of a national administrative database to guide public decisions: From the *système national d’information interrégimes de l’Assurance Maladie* (SNIIRAM) to the *système national des données de santé* (SNDS) in France. Rev Épidémiol Sté Publique. 2017;65:S149–67. 10.1016/j.respe.2017.05.004 28756037

[45] SNDS - regulations, processing, information to data subjects. EPI-PHARE. https://www.epi-phare.fr/en/regulation-snds/ (accessed 28 Dec 2024).

[46] Rey G, Jougla E, Fouillet A, Hémon D. Ecological association between a deprivation index and mortality in France over the period 1997 – 2001: variations with spatial scale, degree of urbanicity, age, gender and cause of death. BMC Public Health. 2009;9:33. 19161613 10.1186/1471-2458-9-33 PMC2637240

[47] Rachas A, Gastaldi-Ménager C, Denis P, Barthélémy P, Constantinou P, Drouin J, et al. The Economic Burden of Disease in France From the National Health Insurance Perspective: The Healthcare Expenditures and Conditions Mapping Used to Prepare the French Social Security Funding Act and the Public Health Act. Med Care. 2022;60:655–64. 35880776 10.1097/MLR.0000000000001745 PMC9365254

[48] l’Assurance Maladie. Méthode de la cartographie des pathologies et des dépenses de l’Assurance Maladie. https://www.assurance-maladie.ameli.fr/etudes-et-donnees/par-theme/pathologies/cartographie-assurance-maladie/methode-cartographie-pathologies-depenses-assurance-maladie (accessed 30 Dec 2024).

[49] Semenzato L, Botton J, Drouin J, Cuenot F, Dray-Spira R, Weill A, et al. Chronic diseases, health conditions and risk of COVID-19-related hospitalization and in-hospital mortality during the first wave of the epidemic in France: a cohort study of 66 million people. Lancet Reg Health - Eur. 2021;8:100158. 34308411 10.1016/j.lanepe.2021.100158 PMC8282330

[50] Rousseau A, Michiels S, Simon-Tillaux N, Lolivier A, Bonastre J, Planchard D, et al. Impact of pembrolizumab treatment duration on overall survival and prognostic factors in advanced non-small cell lung cancer: a nationwide retrospective cohort study. Lancet Reg Health – Europe **43**(2024) 10.1016/j.lanepe.2024.100970. 10.1016/j.lanepe.2024.100970 PMC11262181 39040528

[51] Li J. The exploration of the approach to data preparation for Chinese text analysis based on R Language. OALib. 2021;08:1–8.

[52] A Grammar of Data Manipulation. https://dplyr.tidyverse.org/ (accessed 30 Dec 2024).

[53] ggplot2: Elegant Graphics for Data Analysis (3e). https://ggplot2-book.org/ (accessed 30 Dec 2024).

[54] Therneau TM, until 2009) TL (original S->R port and R maintainer, Elizabeth A, Cynthia C. survival: package for Survival Analysis. (2024) https://cran.r-project.org/web/packages/survival/index.html (accessed 30 Dec 2024).

[55] Partridge AH, Hughes ME, Warner ET, Ottesen RA, Wong Y-N, Edge SB, et al. Subtype-dependent relationship between young age at diagnosis and breast cancer survival. J Clin Oncol. 2016;34:3308–14. 27480155 10.1200/JCO.2015.65.8013

[56] Jenkins EO, Deal AM, Anders CK, Prat A, Perou CM, Carey LA, et al. Age-specific changes in intrinsic breast cancer subtypes: a focus on older women. Oncologist. 2014;19:1076–83. 25142841 10.1634/theoncologist.2014-0184 PMC4200998

[57] Wooster R, Neuhausen SL, Mangion J, Quirk Y, Ford D, Collins N, et al. Localization of a breast cancer susceptibility gene, BRCA2, to Chromosome 13q12-13. Science. 1994;265:2088–90. 8091231 10.1126/science.8091231

[58] Galvin A, Courtinard C, Bouteiller F, Gourgou S, Dalenc F, Jacot W, et al. First-line real-world treatment patterns and survival outcomes in women younger or older than 40 years with metastatic breast cancer in the real-life multicenter French ESME cohort. Eur J Cancer. 2024;196:113422. 37977105 10.1016/j.ejca.2023.113422

[59] Schaefers C, Seidel C, Bokemeyer F, Bokemeyer C. The prognostic impact of the smoking status of cancer patients receiving systemic treatment, radiation therapy, and surgery: A systematic review and meta-analysis. Eur J Cancer. 2022;172:130–7. 35763872 10.1016/j.ejca.2022.05.027

[60] Garassino MC, Oskar S, Arunachalam A, Zu K, Kao Y-H, Chen C, et al. Real-world treatment patterns and outcomes of first-line immunotherapy among patients with advanced nonsquamous NSCLC Harboring BRAF, MET, or HER2 alterations. JTO Clin Res Rep. 2023;4:100568. 37744307 10.1016/j.jtocrr.2023.100568 PMC10514206

[61] Machado MAÁ, de Moura CS, Chan K, Curtis JR, Hudson M, Abrahamowicz M, et al. Real-world analyses of therapy discontinuation of checkpoint inhibitors in metastatic melanoma patients. Sci Rep. 2020;10:14607. 32884119 10.1038/s41598-020-71788-z PMC7471311

[62] Appukkuttan S, McManus HD, Liao N, Curry L, Andres R, Kalayeh B, et al. Real-world discontinuation rates among patients with metastatic castration-sensitive prostate cancer who initiated novel androgen receptor inhibitors: A US claims analysis. JCO Oncol Pr. 2025;21:568.

