# Supplementary material for: Real-world use and survival outcomes of sacituzumab govitecan in metastatic triple-negative breast cancer and hormone receptor-positive/HER2-negative metastatic breast cancer
Source: Br J Cancer. 2026 Feb 5;134(8):1198–208. doi: 10.1038/s41416-026-03346-9 (PMC13036041; doi:10.1038/s41416-026-03346-9)
Supplement: Supplementary file 1 — Supplementary materials [file 41416_2026_3346_MOESM1_ESM.pdf]

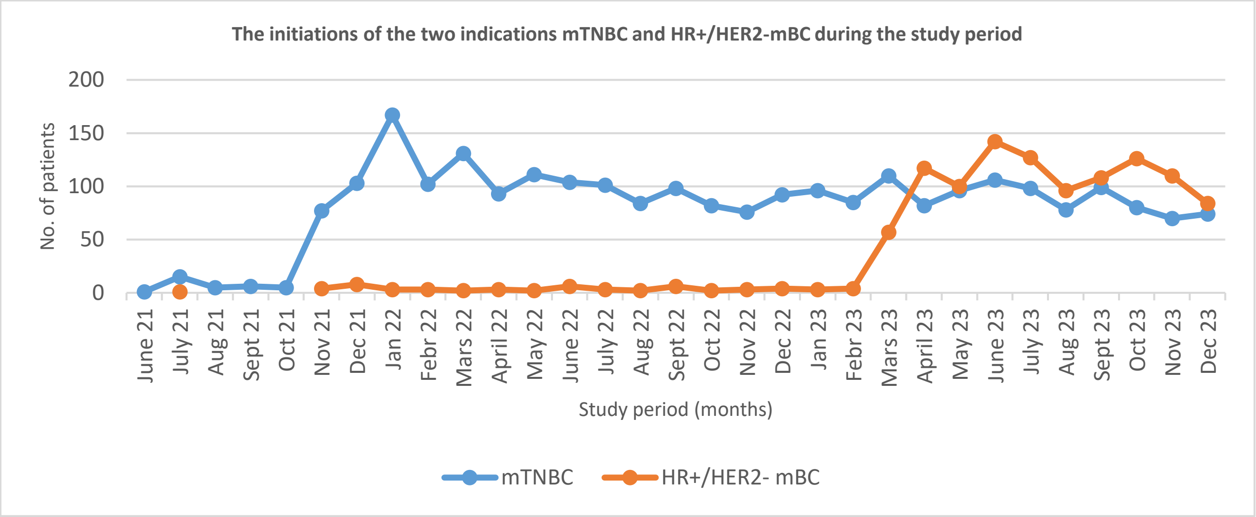

Supplementary figure 1: SG treatment initiations according to the indication, mTNBC or HR+/HER2- mBC during the study period

Supplementary table 1: Below are the UCD (Unité Commune de Dispensation) codes and for Trodelvy® (sacituzumab govitecan) as listed by the Haute Autorité de Santé in France, the Code CIP" stands for "Code Identifiant de Présentation" for Pharmacy dispensation and reimbursement, the Anatomical Therapeutic Chemical Classification System (ATC), developed by the World Health Organization (WHO) and the indication codes (CODE LES for the two early access indications and high cost list indication in mTNBC) from SNDS database. ICD-10 (codes CIM10) are the International Classification of Diseases, 10th Revision codes.

Item	Code	Description
UCD 180 mg	3400890016643	Trodelvy 180 mg (powder for infusion)
CIP 200 mg	3400955085256	Trodelvy 200 mg (powder for infusion) not strictly a UCD
ATC classification	L01FX17	L01F MONOCLONAL ANTIBODIES AND ANTIBODY DRUG CONJUGATES
Code LES early access for indication mTNBC	CSACI01	Monotherapy treatment for adult patients with unresectable or metastatic triple-negative breast cancer who have previously received two or more lines of systemic therapy, including at least one in the advanced stage (ANSM/HAS 02/09/2021)
Code LES early access for indication HR+/HER2- mBC	CSACI02	As monotherapy for the treatment of adult patients with hormone receptor-positive (HR-positive), HER2-negative (IHC 0, IHC 1+ or IHC 2+/ISH-) unresectable or metastatic breast cancer who have received at least two lines of chemotherapy in the metastatic setting (ANSM/HAS 23/02/2023)
Code LES Hight cost list of drugs (liste en sus) in mTNBC indication	I000663	As monotherapy for the treatment of adults with unresectable or metastatic triple-negative breast cancer (TNBC) who have received at least two prior systemic therapies, including at least one for advanced disease (ANSM/HAS 22/08/2024)
Breast cancer diagnosis ICD-10 codes	C50, D05	Specific breast cancer ICD-10 codes (**International Classification of Diseases, 10th Revision** codes) identified in Principal, related and associated diagnosis.
Metastases ICD-10 diagnostic codes	C770, C771, C772, C774, C775, C778, C779, C78, C79, C80	Metastases diagnostic ICD-10 codes (except for the invasive axillary ganglionic metastases C773)

Overall survival (OS)

Time to Treatment Discontinuation (TTD)

Age <=65 vs. >65

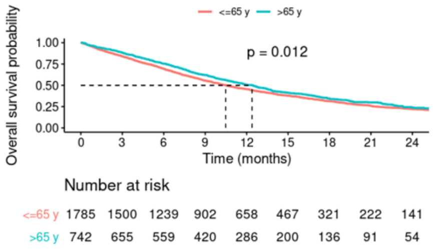

Median OS (months), IC 95%: age <=65: 10.5 [9.9-11.2]
>65: 12.4 [11.2-13.4]
HR: >65 vs <=65
0.87 [0.78-0.97]

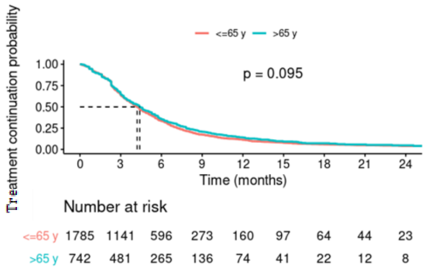

Median TTD (months), IC 95%: age <=65: 4.2 [4-4.4]
>65: 4.4 [4.1-4.7]
HR: >65 vs <=65
0.93 [0.85-1.01]

Number of organs involved in metastasis

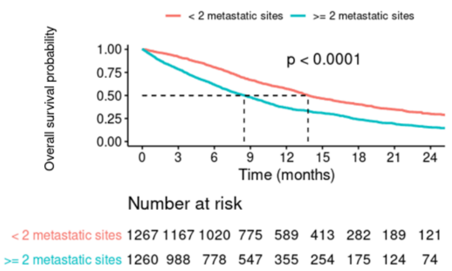

Median OS (months), IC 95%: no. of metastasis sites:
<2 metast sites: 13.8 [13.2-14.8]
>=2 metast sites: 8.5 [7.8-9.2]
HR: >=2 vs <2
1.71 [1.55-1.88]

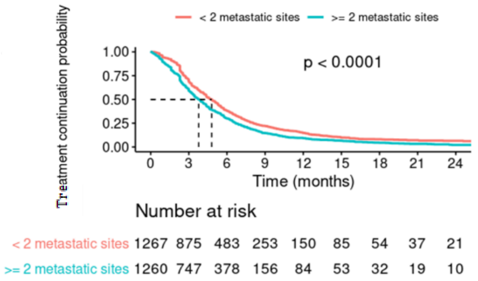

Median TTD (months), IC 95%: no. of metastasis sites:
<2 metast sites: 4.8 [4.4-5.1]
>=2 metast sites: 3.8 [3.5-4.1]
HR: >=2 vs <2
1.3 [1.19-1.41]

Brain Metastasis

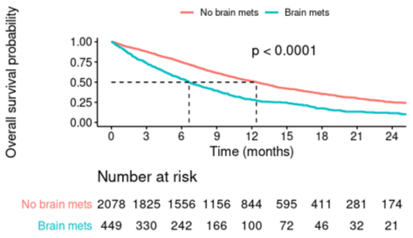

Median OS (months), IC 95%: No BM: 12.4 [11.7-13]
BM: 6.6 [5.8-7.6]
HR: BM vs no BM
1.82 [1.62-2.05]

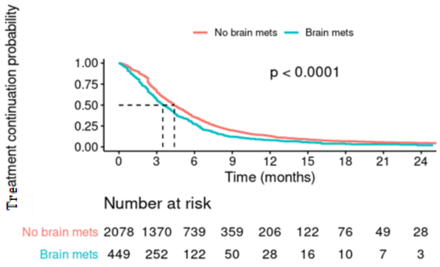

Median TTD (months), IC 95%: No BM: 4.4 [4.2-4.6]
BM: 3.5 [3.1-4]
HR: BM vs no BM
1.3 [1.17-1.45]

Liver Metastasis

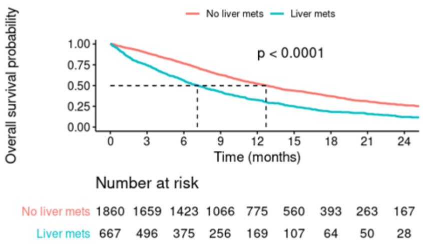

Median OS (months), IC 95%: No LivM: 12.7 [11.9-13.5]
LivM: 7.1 [6.4-8]
HR: LivM vs no LivM
1.77 [1.6-1.96]

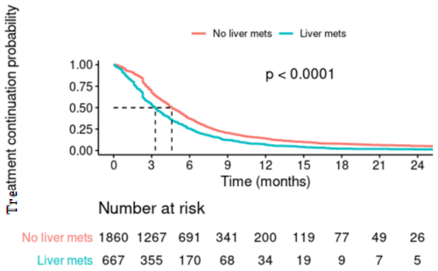

Median TTD (months), IC 95%: No LivM: 4.6 [4.4-4.8]
LivM: 3.3 [3-3.5]
HR: LivM vs no LivM
1.46 [1.33-1.6]

Supplementary figure 2: Metastatic TNBC subgroups survival curves and univariate analysis

Overall survival (OS)

Time to Treatment Discontinuation (TTD)

Age <=65 vs. >65

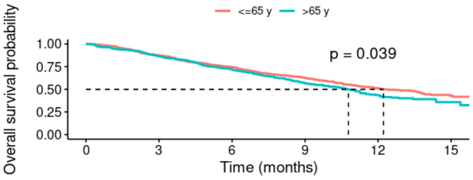

Number at risk

<=65 y	713	624	531	313	151	44
>65 y	413	357	297	166	81	12

Median OS (months), IC 95%: age <=65: 12.2 [11.1-13.9]
>65: 10.8 [9.4-11.9]
HR: >65 vs <=65
1.2 [1.01-1.42]

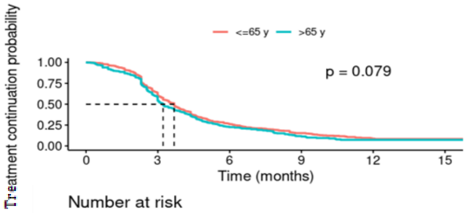

Number at risk

<=65 y	713	431	187	68	21	5
>65 y	413	225	93	27	10	2

Median TTD (months), IC 95%: age <=65: 3.7 [3.5-3.8]
>65: 3.2 [3-3.6]
HR: >65 vs <=65
1.12 [0.99-1.28]

Number of organs involved in metastasis

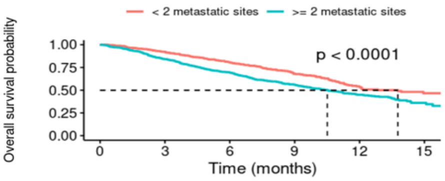

Number at risk

2 metastatic sites	413	381	333	195	94	31
2 metastatic sites	713	600	495	284	138	25

Median OS (months), IC 95%: no. of metastasis sites:
<2 metast sites: 13.8 [11.5-22.4]
>=2 metast sites: 10.5 [9.4-11.8]
HR: >=2 vs <2
1.48 [1.23-1.78]

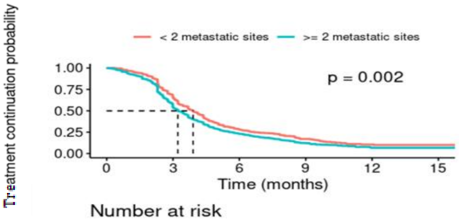

Number at risk

< 2 metastatic sites	413	271	116	43	18	5
>= 2 metastatic sites	713	385	164	52	13	2

Median TTD (months), IC 95%: no. of metastasis sites:
<2 metast sites: 3.9 [3.7-4.3]
>=2 metast sites: 3.2 [3-3.5]
HR: >=2 vs <2
1.23 [1.08-1.4]

Brain Metastasis

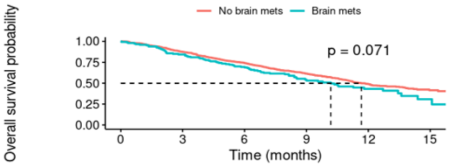

Number at risk

No brain mets	979	857	726	429	208	51
Brain mets	147	124	102	50	24	5

Median OS (months), IC 95%: No BM: 11.7 [11-13.3]
BM: 10.2 [8.4-13.6]
HR: BM vs no BM
1.25 [0.98-1.58]

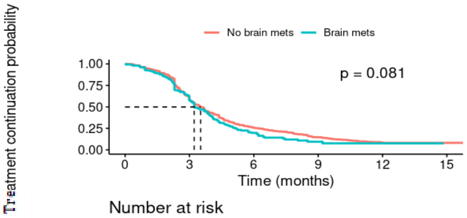

Number at risk

No brain mets	979	571	251	90	29	7
Brain mets	147	85	29	5	2	0

Median TTD (months), IC 95%: No BM: 3.5 [3.2-3.7]
BM: 3.2 [3-3.9]
HR: BM vs no BM
1.18 [0.98-1.41]

Liver Metastasis

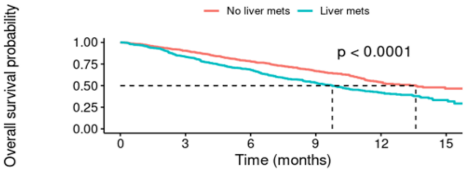

Number at risk

No liver mets	593	536	463	278	127	34
Liver mets	533	445	365	201	105	22

Median OS (months), IC 95%: No LivM: 13.6 [11.9-18.5]
LivM: 9.8 [8.8-11]
HR: LivM vs no LivM
1.5 [1.27-1.78]

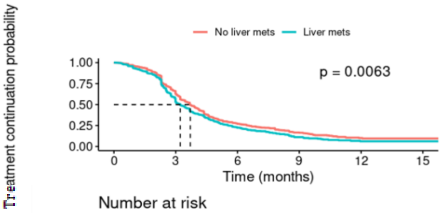

Number at risk

No liver mets	593	373	161	62	20	5
Liver mets	533	283	119	33	11	2

Median TTD (months), IC 95%: No LivM: 3.7 [3.5-4]
LivM: 3.2 [3-3.5]
HR: LivM vs no LivM
1.19 [1.05-1.35]

Supplementary figure 3: Metastatic HR+/HER2- BC subgroups survival curves and univariate analysis

Supplementary table 2: Outcomes before and after adding 116 patients taking hormone therapy within 2 years after initiating by code of indication of mTNBC

Variable	HR+/HER2– (without 116 patients)	HR+/HER2– (with 116 patients)
N	1010 (28.6)	1126 (30.8)
Median age	62 [53;70]	61.5 [53;70]
**Median OS (months), IC 95%**	10.7 m (10.0 – 11.4)	11.4 m (10.7 – 12.6)
Survival rate at 6 months (OS)	71.3% (68.6-74.1)	73.5% (71.0-76.2)
Survival rate at 12 months (OS)	44.5% (40.9-48.3)	48.2% (44.9-51.7)
**Median TTD (months), IC 95%**	3.4 m (3.2 – 3.7)	3.5 m (3.2 – 3.7)
Survival rate at 6 months (TTD)	23.7% (21.6-26.4)	24.9% (22.5-27.5)
Survival rate at 12 months (TTD)	7.9% (6.0-10.4)	8.2% (6.4-10.6)
